# How ecological communities respond to artificial light at night

**DOI:** 10.1002/jez.2157

**Published:** 2018-04-14

**Authors:** Dirk Sanders, Kevin J. Gaston

**Affiliations:** ^1^ Environment and Sustainability Institute University of Exeter Penryn United Kingdom; ^2^ Wissenschaftskolleg zu Berlin Institute for Advanced Study Berlin Germany

**Keywords:** ecosystem functioning, interaction networks, light pollution, species interactions, stability, traits

## Abstract

Many ecosystems worldwide are exposed to artificial light at night (ALAN), from streetlights and other sources, and a wide range of organisms has been shown to respond to this anthropogenic pressure. This raises concerns about the consequences for major ecosystem functions and their stability. However, there is limited understanding of how whole ecological communities respond to ALAN, and this cannot be gained simply by making predictions from observed single species physiological, behavioral, or ecological responses. Research needs to include an important building block of ecological communities, namely the interactions between species that drive ecological and evolutionary processes in ecosystems. Here, we summarize current knowledge about community responses to ALAN and illustrate different pathways and their impact on ecosystem functioning and stability. We discuss that documentation of the impact of ALAN on species interaction networks and trait distributions provides useful tools to link changes in community structure to ecosystem functions. Finally, we suggest several approaches to advance research that will link the diverse impact of ALAN to changes in ecosystems.

## 1 INTRODUCTION

A vast area of the earth is exposed to artificial light at night (ALAN) (Bennie, Davies, Duffy, Inger, & Gaston, 2014; Falchi et al., 2016; Gaston, Duffy, Gaston, Bennie, & Davies, 2014). Such light pollution arises from streetlights and a diversity of other sources typically associated with urban infrastructure. It takes two broad forms, direct light emissions that are typically experienced in close proximity to one or more lamps (e.g., in the vicinity of a streetlight) and skyglow that results from upwardly directed or reflected light emissions being scattered in the atmosphere and creating the familiar artificial brightening of the night sky. Direct light emissions are more intense than skyglow by two or more orders of magnitude (in terms of lux), but narrower in spatial extent by at least an order of magnitude because of the horizontal propagation of skyglow. Virtually all of the major terrestrial ecosystem types experience direct emissions (Bennie, Duffy, Davies, Correa‐Cano, & Gaston, 2015) and thus also skyglow, with the extent of both forms of light pollution growing rapidly, including in previously less economically developed regions of the world (Bennie, Duffy et al., 2015; Kyba et al., 2017).

Although concern about the potential ecological impacts of ALAN has long been expressed (e.g. Cathey & Campbell, 1975; Matzke, 1936; Schroeder, 1945; Verheijen, 1960), fuelled by improved understanding of its regional and global extent, recent years have seen a dramatic increase in research to gain an understanding of these impacts (Davies & Smyth, 2017; Gaston, Visser, & Hölker, 2015). Foremost, this has focused on determining the physiological, behavioral and ecological responses of a number of individual species. This has revealed a range of different pathways by which impacts can occur, particularly through the effects of ALAN on light (and darkness) as a resource (e.g., for photosynthesis, for partitioning of organismal activity between day and night, for dark repair and recovery processes) and on light as a source of information to organisms (e.g., for circadian clocks and photoperiodism, for visual perception, for spatial orientation; Davies, Bennie, Inger, de Ibarra, & Gaston, 2013; Gaston et al., 2015; Gaston, Davies, Nedelec, & Holt, 2017). ALAN can affect a very wide diversity of species. These are already known to include microbes (Hölker et al., 2015), plants (Bennie, Davies, Cruse, & Gaston, 2016; ffrench‐Constant et al., 2016), crustaceans (e.g., Moore, Pierce, Walsh, Kvalvik, & Lim, 1998), insects (e.g., Altermatt & Ebert, 2016; Bird & Parker, 2014; Pacheco‐Tucuch, Ramirez‐Sierra, Gourbière, & Dumonteil, 2012; van Geffen, van Grunsven, van Ruijven, Berendse, & Veenendaal, 2014), spiders (Frank, 2009; Heiling, 1999), fish (Brüning, Hölker, Franke, Preuer, & Kloas, 2015; Riley, Bendall, Ives, Edmonds, & Maxwell, 2012), amphibians (Buchanan, 1993), reptiles (e.g., Thums et al., 2016), birds (e.g., de Jong et al., 2015; Dwyer, Bearhop, Campbell, & Bryant, 2013; Rodríguez et al., 2017), and mammals (e.g., Le Tallec, Perret, & Théry, 2013; Lewanzik & Voigt, 2014; Robert, Lesku, Partecke, & Chambers, 2015).

By contrast with the attention paid to the impacts on individual species, there remains rather limited understanding of how whole ecological communities respond to ALAN. Indeed, a key question must be whether community responses are likely to be predictable from single species responses or if a complementary approach is required. Similar discussions have advanced understanding of the impacts of other anthropogenic environmental impacts, such as climate change (Walther, 2010) and chemical pollution (Maltby et al., 2017).

Often a mechanistic understanding of environmental impacts can be gained from model organisms, an approach that is widely used in biological science (e.g., Hedges, 2002; Scholz et al., 2008). These individual responses can help to predict the impact on a wider range of taxa. However, while very useful to predict other species responses this approach misses an important building block of ecological communities: species interactions. Species in ecological communities form complex networks of direct and indirect interactions and these interactions drive ecological and evolutionary processes in ecosystems. As these networks of interactions are important to predict ecosystem stability and functioning (e.g., Thébault & Fontaine, 2010), a community, rather than single species, approach is necessary for getting a predictive understanding of an overall ALAN impact. Considering the importance of these interaction networks, it has also been suggested to use this information about their structure for biological conservation (Tylianakis, Laliberté, Nielsen, & Bascompte, 2010). Further, biodiversity–ecosystem functioning research has demonstrated a positive relationship between a range of ecosystem functions and species richness (Maestre et al., 2012; Scherber et al., 2010), something that would fail to emerge from single species studies. However, so far studies that look at the impact of ALAN on whole ecological communities are scarce (but see Bennie, Davies, Cruse, Inger, & Gaston, 2015; Davies et al., 2015; Hölker et al., 2015; Knop et al., 2017; Lewanzik & Voigt, 2014), and all of them are limited to certain target taxa or a specialized or particular kind of interaction, rather than looking at the whole community across taxa and functional groups. To get a full understanding of how ALAN impacts ecosystem functions and stability needs a community and ecosystem level approach.

In this paper, we summarize current knowledge about community responses to ALAN and illustrate different pathways that can lead to these responses and their impact on ecosystem functioning and stability. Further, we suggest different approaches as to how best to advance research in this area to meet the outlined challenges.

## 2 HOW ARE ECOLOGICAL COMMUNITIES AFFECTED?

### 2.1 From interactions to networks

An ecological community is defined as the organisms living in the same place at the same time, which may interact in different ways. Core concepts of community ecology include direct and indirect species interactions that can be depicted in food webs or interaction networks. Direct interactions are those between two network nodes or species whereas indirect interactions involve at least a third species to transmit the effect, including, for example, trophic cascades, leading to changes in other parts of the network. Indirect interactions can be very powerful in driving coevolution (Guimarães Jr, Pires, Jordano, Bascompte, & Thompson, 2017) and stability of communities, with their loss triggering extinction cascades (Sanders, Kehoe, & van Veen, 2015). Interactions between organisms are diverse, leading to multilayer networks including different types of interactions, such as consumption (predation, herbivory), parasitism, pollination, competition, and non‐trophic interactions, such as behavior changes or ecosystem engineering (Pilosof, Porter, Pascual, & Kéfi, 2017). The structure of networks and distribution of interaction strength have been used to link these to function and stability (Rooney & McCann, 2012; Tang, Pawar, & Allesina, 2014; Thébault & Fontaine, 2010).

**Figure 1:**
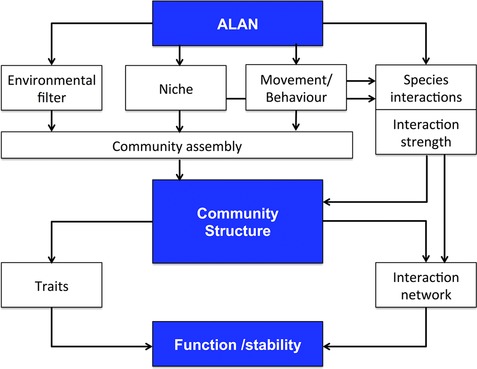
Linking ALAN impact to the stability and functioning of ecological communities. ALAN can act as an environmental filter through a change in animal movement (attraction, avoidance) and behavior or survival thereby influencing community assembly. It can also change communities by changing species’ realized niches and interactions between species. ALAN can increase or decrease interaction strength or lead to new interactions or the extinction of former interactions, thereby changing the structure of interaction networks. A change in community structure can lead to a change in the distribution of traits in communities such as body size or trophic niche. Therefore a major impact of ALAN on function and stability will be through a change in the distribution of species traits and the structure of the interaction network [Color figure can be viewed at http://wileyonlinelibrary.com]

**Figure 2:**
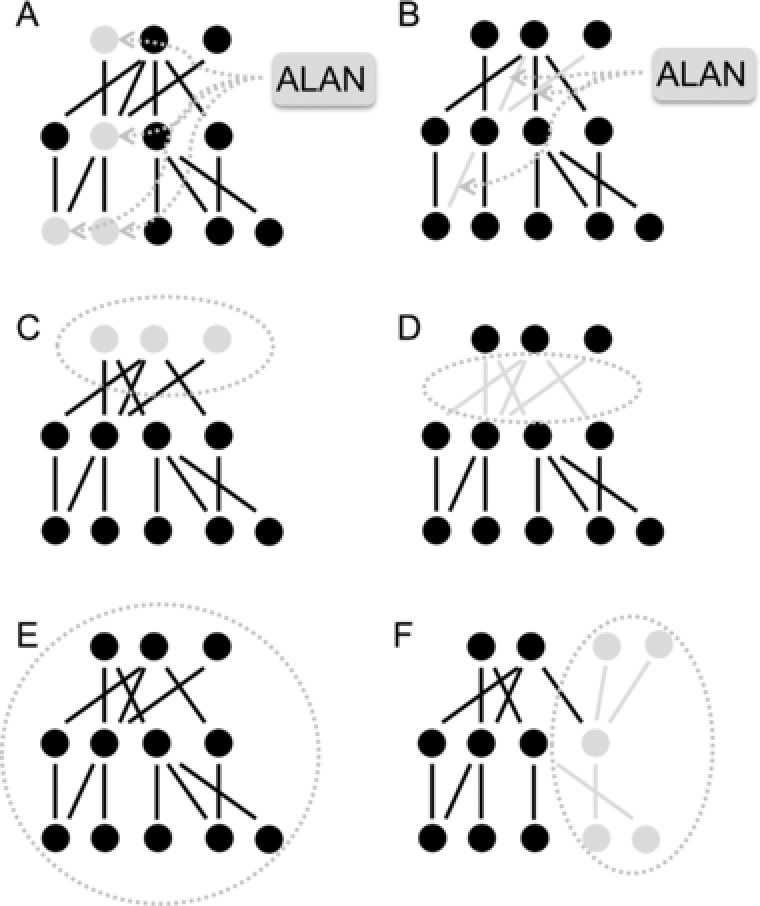
Impact of ALAN on (A) species (nodes) and (B) interactions (links) and different scenarios for different parts in a food web/network affected: (A) single species, (B) single interactions, (C) single trophic levels, (D) interaction between two trophic levels, (E) the whole food web, or (F) a food web or network compartment

An approach to understand the impact of ALAN on ecological communities and their functions needs ultimately to target such interaction networks which includes the impact on network nodes (changes in species growth and survival) and/or interactions between species (Figure 1, Figure 2A, B). Due to the interconnectedness of species, we can expect that even if only a part of the network, such as (i) single species or interactions or (ii) trophic or functional groups, are affected (Figure 2) this will have consequences for the rest of the network unless the impact is contained in a smaller compartment which has only weak links to the wider network (see Figure 2F). For example, if organisms that are active during the dark period respond to ALAN impact by changing their behavior, there will be knock‐on effects on other organisms active during the day if these are connected in a network through direct and/or indirect interactions (such as day and nighttime pollinators, see Knop et al., 2017) or across different habitats when light polluted and non‐polluted areas are linked by movement of individuals and species interactions. A number of studies have looked at the response of ecological communities to artificial light at night. The most well‐known impact is the attraction of aerial invertebrates to artificial lighting through flight‐to‐light behavior (e.g., Verheijen, 1960; van Langevelde, Ettema Donners, WallisDeVries, & Groenendijk, 2011). An attraction to artificially lit areas has also been shown for ground‐dwelling invertebrates, such as ground beetles and spiders. Interestingly, ground‐dwelling invertebrates attracted to lit habitats at night appear not to re‐disperse during the daytime (Davies et al., 2017; Davies, Bennie, & Gaston, 2012; Manfrin et al., 2017) leading to temporally persistent changes in community structure. Similarly, larger bodied predatory fish and small shoaling fish also increased in experimentally lit areas (Becker, Whitfield, Cowley, Järnegren, & Næsje, 2013). These studies suggest that predators in particular appear attracted to lit areas, which are often assumed to follow higher prey abundances (Becker et al., 2013; Davies et al., 2012; Meyer & Sullivan, 2013), potentially leading to higher predation rates and top‐down control. It is, for example, well known that aggregations of invertebrates at lights can in turn attract some species of bats (Rydell, 1991). However, an aggregation of generalist predators will most likely increase intraguild predation which depends strongly on encounter rate between different predators and this has been shown to reduce community‐wide top‐down control (Finke & Denno, 2005).

There is strong evidence that ALAN is changing interactions between species, which might prove to be the most influential overall impact on communities, as has been suggested for the impact of climate change (Alexander, Diez, & Levine, 2015; Ockendon et al., 2014). Evidence for this comes from studies looking at bottom‐up effects in grassland communities (Bennie, Davies et al., 2015) and experimental insect communities (Sanders, Kehoe, Tiley et al., 2015) demonstrating that plant responses to light lead to either increased or reduced plant biomass as well as a different allocation of resources cascading up the food chains. Similarly, this might occur for microbial communities (Hölker et al., 2015) where ALAN impact has been found to lead to a shift to more photoautotrophic bacteria (Cyanobacteria), although another study demonstrated the opposite effect (Grubisic et al., 2017). Also the strength of predator‐prey interactions between ladybirds and aphids has been shown to be influenced by exposure to ALAN, with the magnitude of the change depending on temperature (Miller et al., 2017). A study looking into the foraging behavior of Sowell's short‐tailed bats *Carollia sowelli*, a specialist on the infructescences of pepper plants, concluded that feeding and therefore seed dispersal are negatively affected by ALAN (Lewanzik & Voigt, 2014).

Changes in species interactions should impact population dynamics, which has been shown by Sanders, Kehoe, Tiley et al. (2015). Experimental multigenerational plant‐host–parasitoid communities were exposed to ALAN, which led to major changes in the dynamics of aphids and their parasitoids, resulting in reduced aphid and parasitoid densities. One aphid species *Megoura viciae* responded to ALAN exposure with a larger proportion of the population not switching to sexual reproduction in autumn.

The only study that has so far used the extensive tools provided by network analysis has determined the impact of ALAN on flower visitation networks (Knop et al., 2017). This study documented changes in network structure between a wide range of insects and meadow flowers with reduced flower visitation and pollination, and implications for reduced stability. Nighttime pollinators, which can be very important in some ecosystems, appear especially vulnerable to ALAN impact (Knop et al., 2017; Macgregor et al., 2017) with flight‐to‐light behavior or avoidance of lit areas causing major disruption of pollination services.

### 2.2 Community assembly and niche

ALAN can also act as an environmental filter by changing community assembly. Davies, Coleman, Griffith, and Jenkins (2015) show that night‐time lighting changed the composition of epifaunal marine invertebrate communities. Experimental lighting inhibited or encouraged the colonization of 39% of all taxa analyzed, including sessile and mobile species, leading to different community composition between lit and control sites. ALAN has also been shown to impact the niche space of species by extending the perceived day length and therefore activity patterns for diurnal species (Gaston et al., 2017). How significant this might be for community structure will depend on the extent to which species partition time during day and night for activity because of phylogenetic constraints, and the extent to which they do so to avoid or reduce competition or predation (which might mask underlying circadian rhythms). Whilst it has been argued that phylogenetic constraint plays the major role (e.g. Kronfeld‐Schor & Dayan, 2003), and that switching of the time of activity is rather rare, evidence of the latter continues to accrue, and highlights potential adaptive plasticity (e.g., Hut, Kronfeld‐Schor, van der Vinne, & De la Iglesia, 2012).

### 2.3 Trait distributions in communities

**Figure 3:**
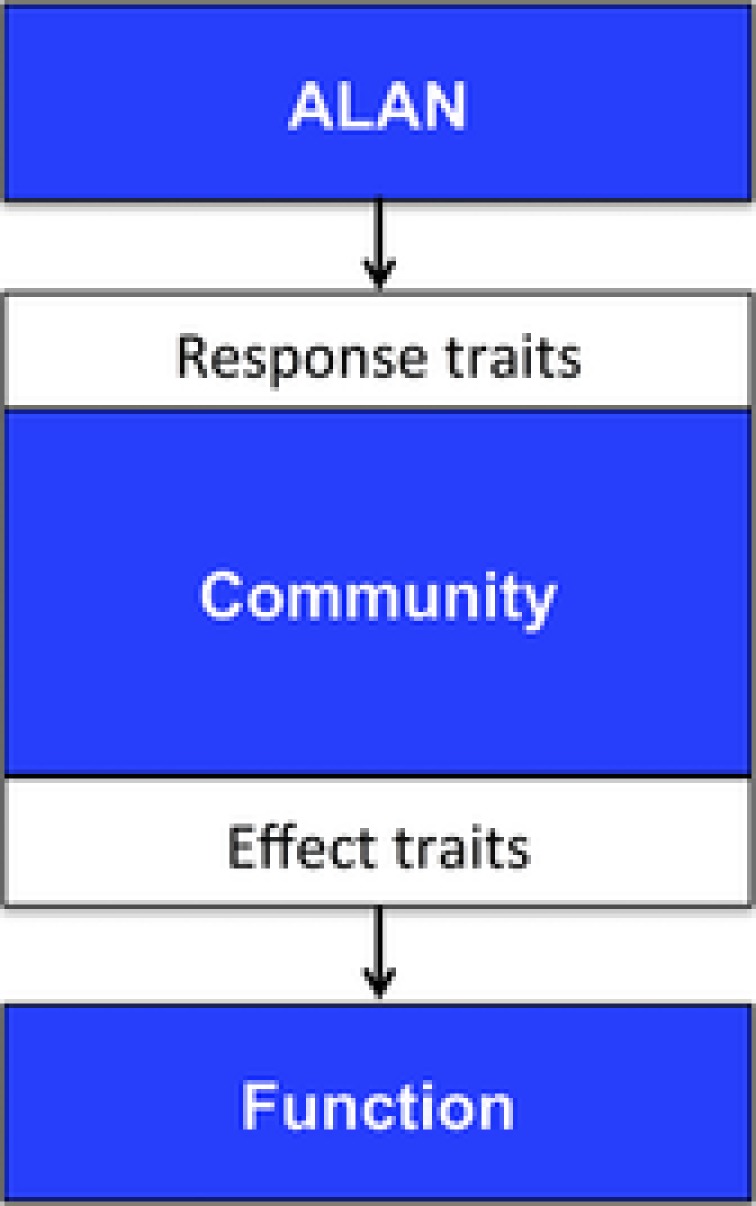
Trait analysis for ecological communities can help to explain their response to ALAN as an environmental factor and how changes in the community are related to changes in ecosystem functions [Color figure can be viewed at http://wileyonlinelibrary.com]

Another useful concept in community ecology is that of species traits and how these are related to environmental pressures and functions (McGill, Enquist, Weiher, & Westoby, 2006). A trait is thereby defined as any morphological, physiological, or phenological feature measurable at the individual level, from the cell to the whole‐organism level (Violle et al., 2007). Traits at the individual level such as body size or size of mouth parts have been used to link communities to functions, such as herbivory (Ibanez, Lavorel, Puijalon, & Moretti, 2013) and trophic niche size in predators (Sanders, Vogel, & Knop, 2015), but very often trait measures are assumed at the species or even functional group level. A useful refinement of the trait concept is to differentiate between (i) response traits that attribute a response to changes in environmental conditions and (ii) effect traits that are linked to community or ecosystem properties (Figure 3). Response traits such as flight‐to‐light behavior or daily activity patterns can, for example, be used to predict how communities should be restructured by ALAN impact and by knowing how certain traits such as specialism–generalism are linked to a role in the community these can then in turn link this to ecosystem functioning. One study found that ALAN strongly influenced multiple invertebrate responses in riparian habitats and also changed the distribution of traits within the exposed communities (Meyer & Sullivan, 2013). In this study an experimental light addition resulted in a 44% decrease in tetragnathid spider density and an overall decrease of 16% in spider family richness. The authors also looked at the distribution of traits and discovered a 76% increase in mean body size of aquatic emergent insects, and a 309% increase in mean body size of terrestrial arthropods. Therefore the community wide impact of ALAN can go in both directions by increasing or decreasing density and/or diversity of organisms and can change the distribution of major traits.

There is evidence that ALAN is affecting network structure and trait distributions with consequences for ecosystem functioning, such as fluxes of organisms and nutrients between terrestrial and aquatic systems, plant biomass, top‐down control, pollination and seed dispersal. A community approach that includes measuring ecosystem functions will allow uncovering how ALAN is changing ecosystem stability and functions (Figure 1) and lead to better predictive understanding of ecosystem wide ALAN effects. Findings from single species studies, while very useful, have the potential to be misleading in terms of impacts on communities, where interactions between species are of major importance.

## 3 FUTURE DIRECTIONS TO INCLUDE COMMUNITY ECOLOGY INTO ALAN RESEARCH

As outlined above, we think that a focus on whole communities by looking at interaction networks and trait distributions will greatly improve understanding of ALAN effects on ecosystem functions (Figure 1). First steps in this direction have already been taken (see Bennie, Davies et al., 2015; Davies et al., 2015; Knop et al., 2017), but we need studies that cover different habitats and interfaces between habitats, that acknowledge ALAN as a diverse impact (see below), and that specifically measure functions and stability and how changes in communities bring these impacts about. We outline five different areas that we think will lead to a better understanding of how ALAN affects ecosystem functions.

1. Empirical research using a combination of observation and experiments needs to target major players for ecosystem functions and measure the impact of ALAN on these functions. A focus should be on community responses in terms of changes in food web or network structure and/or distribution of traits. These changes also need to be linked to the wider community (e.g., nocturnal–diurnal components, as done for pollinators in Knop et al., 2017) to uncover knock on effects.
2. A change in community structure observed under ALAN is potentially the result of one or both of two processes being influenced: community assembly and the structure of naïve communities (Figure 1). Experiments are an excellent tool to uncover the relative importance of these two processes leading to a realized community structure under ALAN. This can be done by looking at the assembly of newly created habitats vs. the naïve communities exposed to ALAN and communities in open vs. closed (caged) plots with the latter preventing immigration of individuals.
3. Considering the large scale impact of ALAN we can expect some adaptations to this environmental pressure, such as a reduction in flight‐to‐light behavior in moths (Altermatt & Ebert, 2016), and this should have consequences for ecosystem functions and might reduce the negative impact of ALAN on these (such as reduction in pollination) to at least some degree. This needs a mechanistic approach by uncovering adaptations in species from ALAN exposed communities and the demonstration that these adapted species perform differently in lit habitats with improved ecosystem functions as compared to naïve exposed communities.
4. It has become obvious that artificial light at night is a diverse impact with a range of different light intensities and spectra that differ in timings and can interact with different day length regimes and seasons. The significance of this variation has so far been little explored (but see Davies et al., 2017; Day, Baker, Schofield, Mathews, & Gaston, 2015; de Jong et al., 2016; Spoelstra et al., 2017). The distribution of ALAN from global to very patchy and small scale impact which also included other components such as variation in intensity and spectrum needs to be included in study designs. Particularly, we expect the impact of skyglow to be very different as compared to direct light emissions, such as in the immediate vicinity of a streetlight. While the latter will very much shift the distribution of organisms by movement (attraction and avoidance), the impact of the former happens on a much larger scale and there is no escape. This has to be targeted by different approaches in study design, for example, so that there is no avoidance possible for the organisms under skyglow or large scale ALAN impact—this needs to be reflected in studies by either caged communities or creating large scale light treatments. This impact will vary with mobility of the organisms (e.g., flying vs. crawling).

It would further be very interesting to compare ALAN impacts in temperate to tropical areas. One might argue that a response should be stronger in temperate areas, because organisms are known to respond to variation in daylength as this is a crucial trigger for certain stages of their lifecycle. But, evidence shows that tropical species are able to detect very small seasonal variations in day length (e.g., Hau, Wikelski, & Wingfield, 1998; Rivera et al., 2002), and therefore it is possible that they are very sensitive to ALAN impact.

5. Finally, we need research that combines different human impacts to test for interactive effects (e.g. disentangle impact of urbanization) (Gaston et al., 2014). This will lead to a better understanding of how mitigation strategies that can potentially more easily target reduction in ALAN (Gaston, Davies, Bennie, & Hopkins, 2012) will remove pressure from ecological communities. There have so far only been a few studies, for example, by combining ALAN and temperature increase to examine predator–prey interactions with implications for biological control under climate change scenarios (Miller et al., 2017), and combining light and sound to examine parasitism in frogs (McMahon, Rohr, & Bernal, 2017) and singing behavior of robins (Fuller, Warren, & Gaston, 2007).
